# A Novel Joint Gene Set Analysis Framework Improves Identification of Enriched Pathways in Cross Disease Transcriptomic Analysis

**DOI:** 10.3389/fgene.2019.00293

**Published:** 2019-04-12

**Authors:** Wenyi Qin, Xujun Wang, Hongyu Zhao, Hui Lu

**Affiliations:** ^1^Center for Biomedical Informatics, Shanghai Children's Hospital, Shanghai Jiaotong University, Shanghai, China; ^2^Department of Bioengineering, University of Illinois at Chicago, Chicago, IL, United States; ^3^Department of Genetics, School of Medicine, Yale University, New Haven, CT, United States; ^4^Department of Bioinformatics and Biostatistics, SJTU-Yale Joint Center for Biostatistics, Shanghai Jiaotong University, Shanghai, China; ^5^Department of Biostatistics, School of Public Health, Yale University, New Haven, CT, United States

**Keywords:** public data integration, cross disease transcriptome, gene expression, gene set enrichment analysis, mixture model, EM algorithm

## Abstract

**Motivation:** Gene set enrichment analysis is a widely accepted expression analysis tool which aims at detecting coordinated expression change within a pre-defined gene sets rather than individual genes. The benefit of gene set analysis over individual differentially expressed (DE) gene analysis includes more reproducible and interpretable results and detecting small but consistent change among gene set which could not be detected by DE gene analysis. There have been many successful gene set analysis applications in human diseases. However, when the sample size of a disease study is small and no other public data sets of the same disease are available, it will lead to lack of power to detect pathways of importance to the disease.

**Results:** We have developed a novel joint gene set analysis statistical framework which aims at improving the power of identifying enriched gene sets through integrating multiple similar disease data sets. Through comprehensive simulation studies, we demonstrated that our proposed frameworks obtained much better AUC scores than single data set analysis and another meta-analysis method in identification of enriched pathways. When applied to two real data sets, the proposed framework could retain the enriched gene sets identified by single data set analysis and exclusively obtained up to 200% more disease-related gene sets demonstrating the improved identification power through information shared between similar diseases. We expect that the proposed framework would enable researchers to better explore public data sets when the sample size of their study is limited.

## Background

High-throughput technology like microarray and next-generation sequencing (NGS) allows researchers measure the expression levels of thousands of genes or microRNAs in one sample simultaneously. These high-throughput genomic data have enabled researchers to better identification of disease related genes and pathways (Gu et al., 2014, 2017; Zheng et al., 2015, 2016; Liu et al., 2016, 2017, 2018; Gong et al., 2018). Gene set enrichment analysis has become a widely accepted expression analysis tool whose purpose is to identify coherent altered expression change within a predefined gene set or a pathway rather than identifying individual differentially expressed (DE) genes (Mootha et al., 2003; Kim and Volsky, 2005; Subramanian et al., 2005; Nam and Kim, 2008). Compared with DE gene analysis, more reproducible and interpretable results could be obtained through gene set enrichment analysis. Gene set enrichment could also detect small but consistent change which is ignored by DE gene analysis (Luo et al., 2009). There are many successful applications of gene set enrichment analysis approach in human disease-related gene/pathway discovery. For example, Drier et al. (2013) showed that enriched gene sets could serve as biomarkers in predicting survival time in glioblastoma and colorectal cancer patients. Zhao et al. combined gene set enrichment analysis information and microRNA target gene sets to identify cancer-related microRNAs (Zhao et al., 2014). Lee et al. utilized gene set enrichment analysis based on mutation and transcriptional data to identify driver mutation behind breast cancer metastasis (Lee et al., 2016). Identifying the enriched gene set will provide crucial information of molecular functions and mechanisms underlying different diseases.

Many gene set enrichment analysis methods have been developed to identify differentially expressed gene sets with different assumptions and data types (Edgar et al., 2002; Kim and Volsky, 2005; Subramanian et al., 2005; Dinu et al., 2007; Freudenberg et al., 2010; Rahmatallah et al., 2015; Zhao and Li, 2017). These methods focused on the analysis of one single data set, thus cannot make full utilization of the rich amount of public expression data. Further, with the cost of microarray and next generation sequencing technique decreasing and stabilization of the experiment protocol, there are now over 1,000,000 samples deposited in public databases such as Gene Expression Ominus (GEO) (Subramanian et al., 2005), meta-analysis is one way to improve the identification power by integrating data sets of same conditions together (Qin et al., 2016). Shen and Tseng (2010) and Chen M. et al. (2013) both proposed meta gene set enrichment analysis frameworks to integrate public data sets of same biological condition and demonstrated improved identification power. However, these meta-analysis frameworks simplify the model by assuming a simple concordance model: a gene is either differentially in all studies or non-differentially expressed in all studies. This is a reasonable assumption when analyzing the dataset of same biological condition but might be problematic in conditions where there are not many public studies available for this disease.

On the other hand, the joint analysis approach has proven more effective in combining multiple different but similar sources of data than meta-analysis approach. The joint analysis methods developed in other fields of omics data analysis have proven useful in increasing the identification power by borrowing information from other similar diseases (Chen X. et al., 2013; Chung et al., 2014; Wang et al., 2016; Lin et al., 2017). In our previous study, we also demonstrated that our joint analysis framework aiming at DE gene detection is more advantageous than single data set analysis and meta-analysis in both simulation studies and real data cases combining different similar disease data sets (Qin and Lu, 2018).

In this study, we extended our previous joint gene analysis framework to joint gene set analysis framework. Base on the assumption that similar disease tends to share similar disease-related genes and pathways (Carson et al., 2017; Qin and Lu, 2018), we developed two joint gene set analysis frameworks aiming at improving identification power of enriched gene sets by borrowing different levels of information from other similar diseases. Compared with previous joint gene analysis framework, we unified DE gene/pathway statistic modeling through a two-component beta-uniform mixture model of *p*-values and combined the model with normalized Kolmogorov-Smirnov (KS) statistic for joint gene set enrichment analysis. These novel frameworks were then compared with single data set analysis as well as the MAPE framework proposed by Shen and Tseng (2010) in simulation studies while Chen's method is not available from their website (Chen M. et al., 2013). The simulation results demonstrated that our proposed joint analysis framework outperformed all other methods in AUC under different simulation scenarios. When applied to two real data examples, the proposed joint analysis framework could recover most of the enriched gene sets which is identified by single data set analysis and further identified more pathways with better biological interpretability than single data set analysis. These results demonstrated the improved identification power of enriched gene sets of the proposed joint gene set analysis framework by borrowing information through similar diseases.

## Methods

### EM Algorithm Implementation for Joint Gene Set Analysis Framework

To perform joint gene set analysis, we need to first address the issue of modeling DE gene/enriched pathway statistics in a single data set. In this study, *P*-values derived from differential test statistics (for example, two sample *t*-statistic or Kolmogorov–Smirnov (KS) statistic designed for detecting enriched pathways) in a single data set are modeled directly by a beta-uniform two component mixture model as described in Pounds and Morris (2003) where the *p*-values of non-DE genes/non-enriched pathways are assumed to belong to uniform distribution and *p*-values of DE genes/enriched pathways belong to a beta distribution with scale parameter α and 1, i.e., *f*(*p*|*D* = 1) = α*p*^α−1^; *f*(*p*|*D* = 0) = 1, where the categorical variable D represents either DE/enriched or non-DE/non-enriched status of a gene/pathway. The marginal density of *p*-value is thus written as follows:

(1) $$\begin{array}{l} f\left(p\right)=\mathrm{Pr}\left(\text{D}=1\right)\alpha p^{\alpha -1}+\left(1-\mathrm{Pr}\left(\text{D}=1\right)\right) \end{array}$$

where Pr(D = 1) is the percentage of DE genes/enriched pathways in a single data set and αϵ(0, 1) is the parameter of the beta distribution. In the joint analysis framework setup, let ***p***_***g***_ = {*p*_*g*1_, …*p*_*gN*_} represent all computed *p*-values of *g*-th gene/pathway across *N* diseases. The formula (1) could be extended to N diseases:

(2) $$\begin{array}{l} f\left(p_{g}\right)=\underset{\mathrm{Pr}\left(D_{1}\ldots ,D_{N}\right)}{\sum }\mathrm{Pr}\left(D_{1}\ldots ,D_{N}\right)\underset{i=1..N}{\prod }f\left(p_{gi}|D_{i}\right) \end{array}$$

where Pr(*D*_1_…, *D*_*N*_) represents the global configuration of DE gene/enriched pathway status across all diseases. In this model, Pr(*D*_1_…, *D*_*N*_) and **α** = {α_1_,α_2_, …α_*N*_} need to be estimated from the data. This is a typical mixture model problem, therefore an EM algorithm is implemented to obtain the maximum likelihood estimate of these parameters following the derivation in previous literature (Pounds and Morris, 2003; Qin and Lu, 2018). The details are described as follows:

Given initial guess of ${\mathrm{Pr}}^{\left(0\right)}\left(D_{1}\ldots ,D_{N}\right)=\frac{1}{2^{N}}$ and $\alpha^{\left(0\right)}=\left\{\alpha_{1}{}^{\left(0\right)},\alpha_{2}{}^{\left(0\right)}\ldots \alpha_{N}{}^{\left(0\right)}\right\}$ where $\alpha_{i}{}^{\left(0\right)}=0.5\$,$, the EM algorithm update at *t*-th step for **α**^(**t**)^ and Pr(*D*_1_…, *D*_*N*_) is written as follows:

#### E-Step

The posterior probability of *g*-th gene's configuration status given observed ***p***_***g***_ and **α**^(***t***)^ is given by:

(3) $$Pr(D_{1}\ldots ,D_{N}|\;p_{g},\;\alpha^{\left(t\right)})=\frac{f(p_{g}|D_{1}\ldots ,D_{N},\alpha^{\left(t\right)}(D_{1}\ldots ,D_{N})}{f\left(\;p_{g},\alpha^{\left(t\right)}\right)}$$

#### M-Step

Then the updated ${\mathrm{Pr}}^{\left(t+1\right)}\left(D_{1}\ldots ,D_{N}\right)$ and **α**^(**t+1**)^ is shown as follows:

(4) $$\begin{array}{l} {\mathrm{Pr}}^{\left(t+1\right)}\left(D_{1}\ldots ,D_{N}\right)=\frac{\sum_{g=1}^{G}\mathrm{Pr}\left(D_{1}\ldots ,D_{N}|p_{g},\;\alpha^{\left(t\right)}\right)}{G} \end{array}$$

(5) $$\begin{array}{l} {\alpha_{j}}^{\left(t+1\right)}=\frac{\sum_{g=1}^{G}\mathrm{Pr}\left(D_{1}\ldots D_{j}=1,D_{N}|p_{g},\;\alpha^{\left(t\right)}\right)}{\sum_{g=1}^{G}\mathrm{Pr}\left(D_{1}\ldots D_{j}=1,D_{N}|p_{g},\;\alpha^{\left(t\right)}\right)\left(-logp_{gj}\right)} \end{array}$$

### Normalized KS Statistic and Corresponding *p*-value Calculation for a Pathway

Normalized KS statistic defined in Mootha et al. (2003) is used to detect significantly enriched pathways by measuring if the ranks of genes along one pathway are more enriched on the top rank of an ordered gene list than expected by chance while controlling for pathway size. A normalized KS statistic for a pathway *P* containing M members is computed as follows:

1. Order all *G* genes by their statistical significance.
2. Calculate$R_{i}=-\sqrt{\frac{M}{G-M}}$ if the gene *i* does not belong to a pathway; Calculate$R_{i}=\sqrt{\frac{G-M}{G}}$ if the gene *i* belongs to the pathway.
3. Run a running sum across all G genes and compute the normalized KS statistic as:

(6) $$\begin{array}{l} \text{nK}{\text{S}}_{P}=\underset{j=1\;to\;G}{\max}\overset{j}{\underset{i=1}{\sum }}R_{i} \end{array}$$

To evaluate the significance of the observed normKS for a pathway, a gene-based permutation test is used to calculate the *p*-value.

The permutation test contains the following steps:

1. Random permutate the gene labels.
2. Compute the permutated normalized KS statistics for each pathway and pool them together as *nKS*_*perm*_.
3. Repeat step 1 and 2 B times.
4. The *p*-value of a pathway P could be obtained by counting how many permutated normalized KS statistics are larger than the observed normalized KS statistic, i.e.,:
  (7) $$\begin{array}{l} \text{p}\left({\text{nKS}}_{P}\right)=\frac{\sum I\left({\text{nKS}}_{P}\geq {\text{nKS}}_{perm}\right)+1}{B*P+1} \end{array}$$
  where I(·) is the indicator function.

### Gene-Level Joint Gene Set Enrichment Analysis Framework (JointNormKS)

Based on the two-component mixture modeling of *p*-value for a single data set defined before a gene-level joint gene set enrichment framework is then developed which is based on normalized KS statistic (JointNormKS). The outline of the framework could be summarized as follows:

1. Compute and convert the differential statistics into *p*-value, denote *p*_*gi*_ as the *p*-value of gene *g* in data set *i*.
2. Joint analysis based on a two-component beta-uniform mixture model is performed with these *p*-values and the posterior probability of DE status for each gene *g* in disease *i* is computed:
  (8) $$\begin{array}{l} \mathrm{Pr}\left(D_{i}=1|p_{g1},\ldots ,p_{gN}\right)=\\ \frac{\sum_{D_{i}=1}f\left(p_{g1},p_{g2}\ldots ,p_{gN}|D_{1},D_{2},\ldots D_{i}=1,\ldots D_{N}\right)\mathrm{Pr}\left(D_{1},D_{2},\ldots D_{i}=1,\ldots D_{N}\right)}{f\left(p_{g1},p_{g2}\ldots ,p_{gN}\right)} \end{array}$$
3. Compute normKS statistic and corresponding *p*-value based on the ranking of posterior probability Pr(*D*_*i*_ = 1|*p*_*g*1_, …, *p*_*gN*_) within each data set *i*.
4. After p-values of all pathways within each data set are computed, use Benjamini-Hochberg (BH) procedure (Benjamini and Hochberg, 1995) to compute FDR for each pathway and order the pathways within each dataset by the FDR respectively.

### Pathway-Level Joint Pathway Enrichment Analysis Framework (JointPathway)

In this section, JointPathway is proposed as another joint gene set enrichment analysis framework which summarizes the enrichment evidence on pathway-level first within each disease data set and then performs joint analysis on pathway-level *p*-value to identify potential enriched pathways. The assumption of the framework is based on that similar disease tends to share similar shared dysregulated pathways. The outline of the framework is summarized as follows:

1. Within each disease dataset, compute the normKS statistics for each pathway and obtain their *p*-values based on the permutation procedures denoted as ***p***_***gi***_ where *g* represents *g*-th pathway and *i* represents *i*-th disease data set. The implementation of the permutation procedures is described in detail in JointNormKS section.
2. Perform joint analysis procedure based on ***p***_***gi***_ of all pathways across all data sets. Estimate prior probability, Pr(*D*_1_…, *D*_*N*_), and beta distribution parameter of each data set, **α** = {α_1_,α_2_, …α_*N*_}, from ***p***_***gi***_ through EM algorithm as described before.
3. Compute posterior probability Pr(*D*_*i*_ = 1|*p*_*g*1_, …, *p*_*gN*_) of for each pathway *g* within each data set *i* as similarly defined in Equation (8) in JointNormKS and rank the pathways accordingly.

### Meta-Analysis for Pathway Enrichment Analysis (MAPE)

Meta-Analysis for Pathway Enrichment Analysis (MAPE) is a series of meta-analysis frameworks proposed by Shen and Tseng (2010), which is specifically designed for pathway/gene set enrichment meta-analysis. It consists of three different frameworks: MAPE_Gene, MAPE_Pathway, and MAPE_I. Here, we briefly introduce the implementation of each framework.

MAPE_Gene could be summarized by the following steps:

1. Compute *p*-value of differential statistic for each gene.
2. Perform MaxP meta-analysis for all genes across all data sets.
3. Compute KS statistics for each pathway.
4. Determine the *p*-value and false discovery rate (FDR) for each pathway through permutation test.

MAPE_Pathway could be summarized by the following steps:

1. Compute KS statistic and its *p*-value through permutation test for all pathways within each data set.
2. Perform MaxP meta-analysis for all pathways across all data sets.
3. Determine the *p*-value and FDR for each pathway through permutation test.

MAPE_I is a hybridization of MAPE_Gene and MAPE_Pathway frameworks which takes the minimum *p*-value of a pathway obtained through MAPE_Gene and MAPE_Pathway as its test statistic. The *p*-value and FDR of this statistic are then determined through permutation test.

### Simulation Study

To evaluate the effectiveness of the proposed joint gene set analysis frameworks, we performed comprehensive simulation studies. Assume that there is a total of 1,000 DE genes out of 10,000 genes. The expression value of each gene in a sample within each data set is generated as described in our previous study (Qin and Lu, 2018) with different means and variance set for each gene. We further assume that the number of data sets to be jointly analyzed is fixed at *N* = 2 and the number of shared DE genes between two data sets is fixed at 600, 700, 800, or 900, so the DE gene similarity between two data sets are defined as the average shared percentage of DE genes i.e., $\frac{1}{2}\left(Pr\left(D_{2}=1|D_{1}=1\right)+Pr\left(D_{1}=1|D_{2}=1\right)\right)$ would be 60, 70, 80, and 90%. After the gene expression data are generated, we further assume that there is a total of 1,000 pathways each of which contains 50 genes and therefore we would expect to see 5 DE genes within each pathway and any pathway containing more than 5 DE genes would be considered as an enriched pathway. In this simulation study, we set the number of DE genes of an enriched pathway at 10 and 15, respectively. Within each data set, there is a total of 100 enriched pathways. Similar to DE gene similarity definition, we define the shared number of enriched pathways at 60, 70, 80, and 90 between two data sets and consider it as enriched pathway similarity between two diseases. Each pathway is formed by randomly sampling DE and non-DE gene and could be represented by Table 1 where each row represents the enrichment status of a pathway in two data sets and the number in each cell represents how to sample genes from two data sets. Finally, to systematically evaluate the performance of different frameworks, Receiver Operation Curve (ROC) (Fawcett, 2006) is used. Each parameter setup is repeated 30 times and the average Area Under Curve (AUC) is calculated and recorded for each framework.

**Table 1:** Simulation parameter setup under different scenarios.

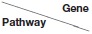	**(0,0)**	**(DE,0)**	**(0,DE)**	**(DE,DE)**
**(A) SCENARIO 1, ENRICHMENT STRENGTH** **=** **20%**				
(0,0)	45	0	0	5
(EP,0)	40	0	5	5
(0,EP)	40	5	0	5
(EP,EP)	40	0	0	10
**(B) SCENARIO 2, ENRICHMENT STRENGTH** **=** **20%**				
(0,0)	45	0	0	5
(EP,0)	40	5	10	0
(0,EP)	40	10	5	0
(EP,EP)	40	0	0	10
**(C) SCENARIO 1, ENRICHMENT STRENGTH** **=** **30%**				
(0,0)	45	0	0	5
(EP,0)	35	0	5	10
(0,EP)	35	5	0	10
(EP,EP)	35	0	0	15
**(D) SCENARIO 2, ENRICHMENT STRENGTH** **=** **30%**				
(0,0)	45	0	0	5
(EP,0)	30	5	15	0
(0,EP)	30	15	5	0
(EP,EP)	20	15	15	0

EP: Enriched Pathway

### Gene Set Collection Database

The up-to-date C2 canonical pathway collection (Version 6.1) of MsigDB (Subramanian et al., 2007) which contains 1,329 gene sets is used in this study. Before the gene set enrichment analysis, any gene set which contains < 15 genes, or more than 500 genes is removed from further analysis.

### Lung Adenocarcinoma and Colorectal Adenocarcinoma

Adenocarcinomas are observed to share similar DE genes as discovered in our previous study (Qin and Lu, 2018), we decide to use lung adenocarcinoma (GEO accession no.: GSE32863) and colorectal adenocarcinoma (GEO accession no.: GSE41258) as one evaluation of our proposed joint gene set analysis frameworks. After we combined multiple probe sets representing same gene by taking the maximum expression value in each sample, a total of 12,054 unique genes and 991 canonical pathways are used in the analysis.

### Alzheimer's Disease (AD) and Huntington's Disease (HD)

AD and HD are known to share highly similar pathology (Narayanan et al., 2014). In this study, GSE33000 which contains both AD and HD postmortem samples are used to evaluate the performance of joint gene set enrichment analysis. Multiple probe sets representing same gene are combined by taking the maximum expression value in each sample. A total of 21,576 genes and 1,071 pathways are used in the analysis.

## Results

### Overview of Proposed Joint Gene Set Enrichment Analysis Frameworks

**Figure 1:**
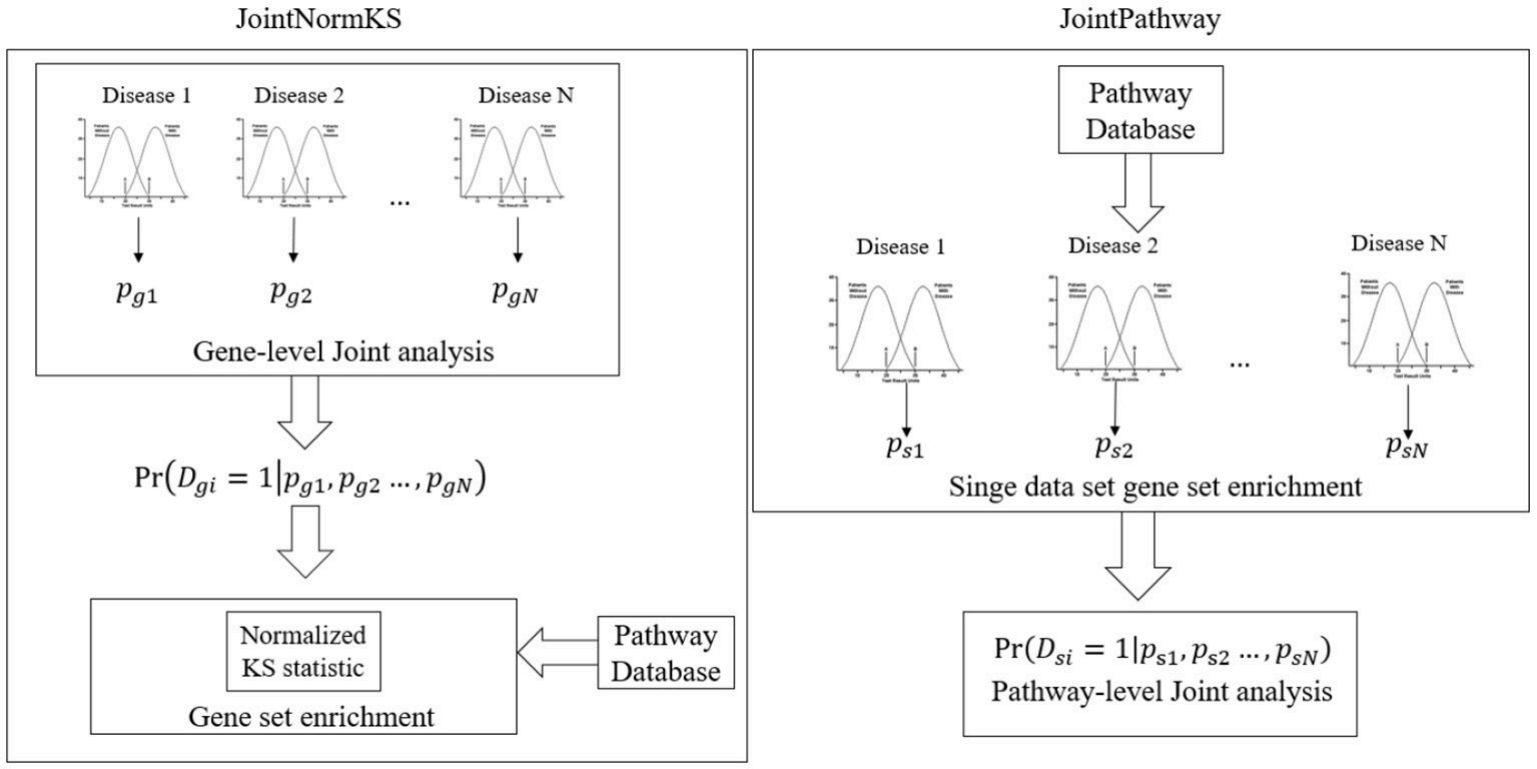
Overview of the proposed joint gene set enrichment frameworks.

Figure 1 outlines the flowchart of three joint gene set enrichment frameworks proposed in this study. The details of the algorithm implementation could be found in the Methods section. Here we briefly discuss the difference between the two frameworks. The joint gene set enrichment framework could be split into gene-level (JointNormKS) and pathway-level (JointPathway). In JointNormKS, the differential expression status of each gene is first jointly analyzed across all similar disease data sets and gene set enrichment analysis is then performed based on the jointly analyzed results which incorporates information from other similar diseases. In this framework, we would expect to observe increased identification power of pathway enrichment when a gene successfully borrows information from other genes. In JointPathway, gene-level information is first summarized based on pathway within each dataset and joint analysis is then performed based on the pathway-level evidence. Under this framework, we would expect to see increased identification power when similar diseases share many enriched pathways among each other.

### Comparisons Among JointNormKS, JointPathway, Single Data Set Analysis and MAPE Methods in Simulated Data Sets

**Figure 2:**
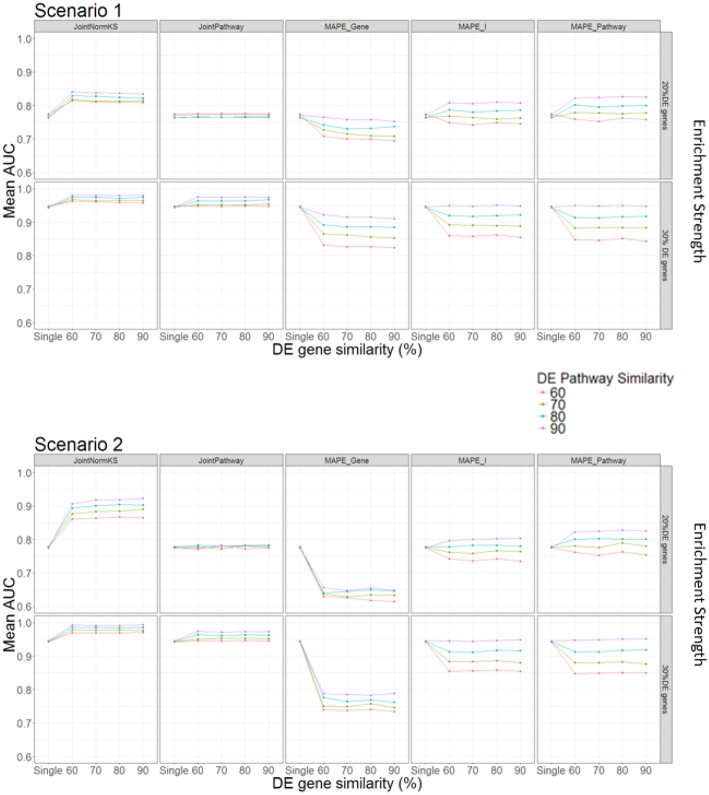
AUC comparison among different methods under different parameter setup.

In this section, we evaluated the performance of the proposed joint gene set enrichment analysis framework through simulation study and compared their performance with single data set analysis and published MAPE methods (Shen and Tseng, 2010). The detailed implementation of the simulation study and parameter setup could be found in Methods section and Table 1. Briefly speaking, expression data sets of two similar diseases are generated with different number of DE genes within a pathway, DE gene similarity and enriched pathway similarity. Furthermore, we consider two different DE gene configuration scenario in the pathway. In the first scenario, the enriched pathway in the target disease data set will contain fully overlapped shared DE genes from the similar disease data set from which information is borrowed. In the second scenario, the DE genes in the enriched pathway of the target disease data set will not overlap with any DE genes in the similar disease data set. This is a reasonable assumption as similar situation has been observed in other literature where one pathway is enriched in both datasets but DE genes are different (Shen and Tseng, 2010). The comparison results are summarized in Figure 2.

In Scenario 1, we assume that one enriched pathway is composed of shared DE genes. In this scenario, we observe that our proposed JointNormKS outperforms all other methods when the enrichment strength is set to 20% DE genes in an enriched pathway. We observe that JointNormKS is not sensitive to the DE gene similarity, different DE gene similarity yields similar significant AUC improvement over single data set analysis. On the other hand, enriched pathway similarity shows a stronger impact on the performance of JointNormKS: the AUC improves when the enriched pathway similarity increases. JointPathway in this scenario does not show difference with single data set analysis when the enrichment strength is low mainly because the *p*-value signals of enriched and non-enriched pathways are not separable in this case. The information borrowing in the joint analysis is thus not working for low-signal case. MAPE methods do not work well in this case. MAPE_Gene shows worse performance in all Enriched pathway parameter setup mainly because when MAPE_Gene summarizes evidence at gene-level, it takes the maximum *p*-value of a gene in both diseases which will lead to failing to identify many disease-specific DE genes in a pathway. MAPE_Pathway shows increased performance when enriched pathway similarity increases. However, even when the enriched pathway similarity is set to 90%, JointNormKS still outperforms MAPE_Pathway because disease-specific pathway will be regarded as false positive by MAPE_Pathway and thus has a low rank. MAPE_I method combines best results calculated from MAPE_Gene and MAPE_Pathway methods and thus cannot demonstrate better performance than JointNormKS. When the enrichment strength increases from 20% DE genes to 30% DE genes, JointNomrKS still outperforms all other methods. we also observe that JointPathway demonstrates improved AUC over single data set analysis when the enrichment strength increases because the signal of an enriched pathway in a single data set could be distinguished from non-Enriched pathway which enables the information sharing between two similar diseases. MAPE_gene performs similar as before while MAPE_pathway does not show improvement over single data set analysis mainly because when the signal of a single data set is strong enough, meta-analysis-based method would, on the contrary, cause the decrease of the rank of disease-specific Enriched pathway.

In Scenario 2, we assume that enriched pathways are composed of non-overlapping DE genes in two data sets. JointNormKS still outperforms all other methods in this scenario. The AUC improvement is even larger than that in scenario 1. As we further examine the result, we find that the reason that JointNormKS could efficiently borrow shared enriched pathway information is due to the combined use of normalized KS statistic and joint analysis at gene level (see Conclusion and Discussions for details). MAPE_Gene performs even worse in this scenario because there is not shared DE genes within a pathway. Meta-analysis by taking maximum *p*-value would thus produce many false positives in DE gene detection. Other methods based on pathway-level evidence summarization remain same performance as in Scenario 1.

To sum up, the simulation test with different parameter setup and two different scenarios demonstrates that JointNormKS performs best among all other methods even when there are no shared DE genes within an enriched pathway. We then decide to use JointNormKS method in real data application in next section.

### Comparison of JointNormKS With Single Data Set Analysis in Real Data Application

Based on the simulation test results, we apply the JointNormKS framework on two real data sets and compare their identified enriched gene sets with those derived from single data set analysis, respectively. We use lung and colorectal adenocarcinoma as one example because adenocarcinoma both develop from gland cells of different tissues and as shown in our previous study, we observed that lung and colorectal adenocarcinoma shared a significant higher percentage of DE genes than other cancers (Qin and Lu, 2018). Alzheimer's disease and Huntington's disease are selected as another example due to their highly similar clinical phenotypes.

#### Real Data Application: Lung Adenocarcinoma and Colorectal Adenocarcinoma

**Figure 3:**
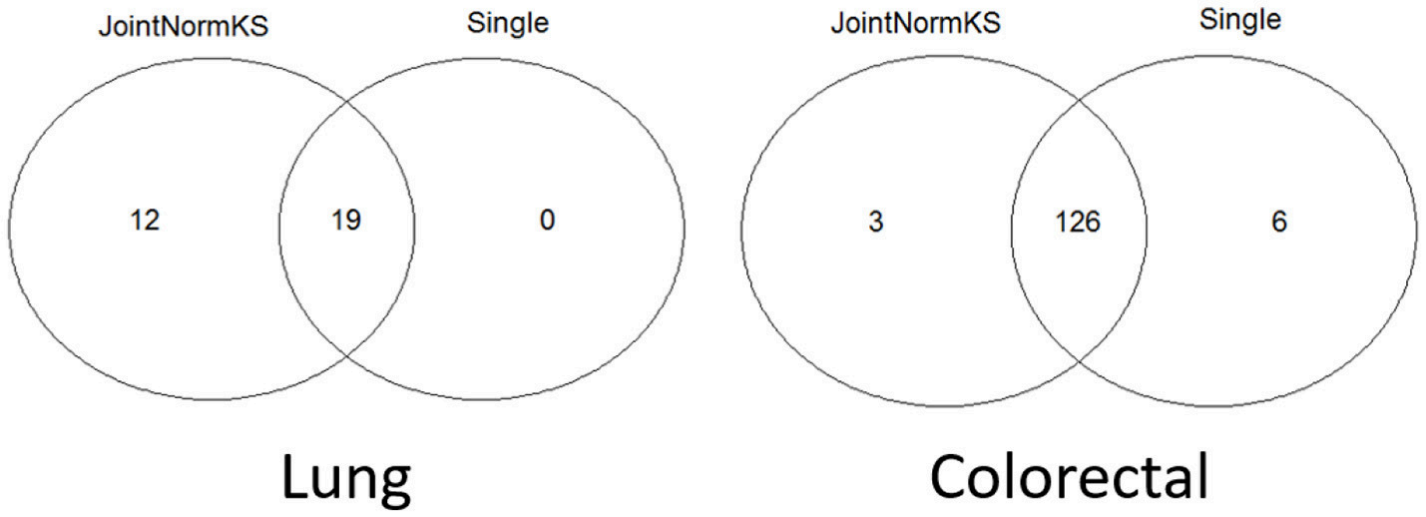
Venn diagram of identified enriched pathways by JointNormKS and single data set analysis in lung and colorectal adenocarcinoma data sets. FDR cutoff is set to 0.1.

JointNormKS is first applied on adenocarcinoma data sets and results are compared with those obtained through single data set analysis with the use of NormKS statistic by setting the FDR cutoff at 0.1. The comparison results are summarized in Figure 3. In lung adenocarcinoma data set, single data set analysis identified 19 pathways while JointNormKS could identify all these pathways plus 12 more enriched pathways. The common pathways identified by both methods contain “KEGG_CELL_CYCLE” which is the KEGG pathway documented in KEGG disease pathway database about known pathways involved with non-small cell lung cancer (pathways taken from hsa05223). The *p*-value and FDR of this pathway is significantly improved in JointNormKS (FDR~0.005) compared with single data set analysis (FDR~0.012). We also examined other known pathways involved with non-small-cell lung cancer recorded in KEGG and found that most of these pathways have improved significance in JointNormKS over single data set analysis (Additional File 1A). Among other commonly identified pathways, many cancer related pathways are identified including cell cycle related pathways such as “REACTOME_DNA_REPLICATION” and cancer signaling pathways such as “PID_E2F_PATHWAY” (Nevins, 2001; Bracken et al., 2003; Tazawa et al., 2007), “PID_AURORA_B_PATHWAY” all of which play an important role in tumor progress (Chieffi et al., 2006; Girdler et al., 2006; Qi et al., 2007). For exclusively identified pathways by JointNormKS shown in Table 2, many of them are related to lung cancer after an extensive literature search. For instance, “PID_MYC_ACTIV_PATHWAY” is a classic cancer-related pathway regulating cell proliferation process which is found in many cancers (Zajac-Kaye, 2001; Bild et al., 2006; Chou et al., 2010). “BIOCARTA_MCM_PATHWAY” which controls initialization of DNA replication process was reported in several lung cancer studies (Ho et al., 2007; Brambilla and Gazdar, 2009). Other pathways which is closely related to cancer progress includes pathways of amino acid metabolism and DNA synthesis. The full list of identified pathways in lung adenocarcinoma could be found in Additional File 1B.

**Table 2:** Pathways exclusively identified by JointNormKS in lung adenocarcinoma data set.

**Pathway**	**Single FDR**	**JointNormKS FDR**
KEGG_BASE_EXCISION_REPAIR	0.1011	0.0797
KEGG_BLADDER_CANCER	0.1144	0.0988
BIOCARTA_MCM_PATHWAY	0.1144	0.0999
BIOCARTA_COMP_PATHWAY	0.1004	0.0797
BIOCARTA_CELLCYCLE_PATHWAY	0.1011	0.0912
PID_MYC_ACTIV_PATHWAY	0.1093	0.0961
PID_AURORA_A_PATHWAY	0.1035	0.0961
REACTOME_MUSCLE_CONTRACTION	0.1144	0.0978
REACTOME_SYNTHESIS_OF_DNA	0.1011	0.0867
REACTOME_METABOLISM_OF_CARBOHYDRATES	0.1144	0.0961
REACTOME_COMPLEMENT_CASCADE	0.1011	0.0797
NABA_ECM_AFFILIATED	0.1144	0.0961

In colorectal adenocarcinoma data sets, single data set analysis slightly identified more enriched pathways than JointNormKS. One hundred and twenty six pathways were identified by both methods. We observe that three pathways are exclusively identified by JointNormKS while six exclusively by single data set analysis. The biological process represented by 126 commonly identified enriched pathways are similar to what was observed in lung adenocarcinoma data set. Among them, “KEGG_CELL_CYCLE” and “KEGG_P53_SIGNALING_PATHWAY” are two pathways that are documented in pathways known to be related to colorectal cancer in KEGG database (hsa05210). When examining all eight pathways known to be related to colorectal cancer, we also observed that JointNormKS overall improved the FDR statistical significance of these pathways compared with single data set analysis. The full result is summarized in Additional File 2A. We further examined the enriched pathways exclusively identified by JointNormKS and single data set analysis, respectively. We find that all three pathways exclusively identified by JointNormKS are closely related to cancer. “BIOCARTA_P53_PATHWAY” and “PID_MYC_PATHWAY” are two canonical cancer-related pathways. As for “REACTOME_TRANSCRIPTION,” after we examined the gene family categorization on MsigDB, we find that many genes in this gene set belong to gene family related to cancer such as “oncogene,” “tumor suppressor” etc. On the other hand, in the six gene sets exclusively identified by single data set analysis, only one gene set: “WNT_SIGNALING” is the process known to be related to cancer progress. The other four gene sets might be potential false positives because very few reports could be found for these biological processes. The full list of identified enriched gene sets in colorectal adenocarcinoma could be found in Additional File 2B.

#### Real Data Application: Alzheimer's Disease and Huntington's Disease

**Figure 4:**
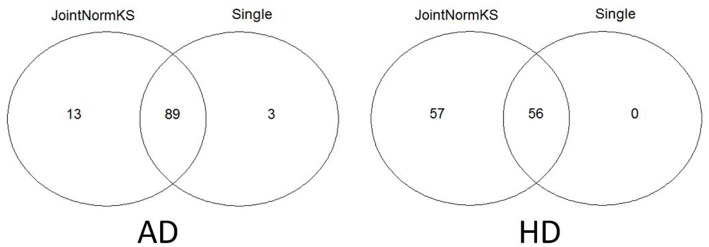
Venn diagram of identified enriched pathways by JointNormKS and single data set analysis in AD and HD data sets. FDR cutoff is set to 0.1.

Furthermore, we apply JointNormKS on two neurodegenerative disorder data sets and evaluate the identified enriched gene sets. The comparison results are summarized in Figure 4. JointNormKS demonstrated improved statistical power by identifying more enriched gene sets than single data set analysis while enriched gene sets identified by single data set analysis could also be identified by JointNormKS. On the other hand, in AD data set, JointNormKS exclusively identified 13 enriched gene sets and in HD data set, the number is 57. A clear statistical power gain is observed in JointNormKS over single data set analysis here.

In AD data set, we first examined three pathways known to be related to AD disease documented in KEGG disease pathway (hsa05010). “KEGG_APOPTOSIS” and “KEGG_OXIDATIVE_PHOSPHORYLATION” are identified by both methods with similar level of significance. The results of three known AD related pathways are summarized in Additional File 3A. A further examination on the 13 exclusively identified gene sets by JointNormKS shows that these gene sets belong to category of apoptosis/cell survival, neuron development and energy metabolism all of which has a close relationship to AD (Table 3). The full list of identified enriched gene sets are summarized in Additional File 3B.

**Table 3:** Pathways exclusively identified by JointNormKS in AD data set.

**Pathway**	**Single FDR**	**JointNormKS FDR**
KEGG_APOPTOSIS	0.0117	0.0087
KEGG_ADIPOCYTOKINE_SIGNALING_PATHWAY	0.0125	0.0090
BIOCARTA_CERAMIDE_PATHWAY	0.0125	0.0096
BIOCARTA_PDGF_PATHWAY	0.0116	0.0081
ST_JNK_MAPK_PATHWAY	0.0128	0.0092
REACTOME_DEVELOPMENTAL_BIOLOGY	0.0268	0.0081
REACTOME_NEURONAL_SYSTEM	0.0128	0.0091
REACTOME_MRNA_PROCESSING	0.0106	0.0087
REACTOME_AXON_GUIDANCE	0.0241	0.0091
REACTOME_REGULATION_OF_MITOTIC_CELL_CYCLE	0.0116	0.0087
REACTOME_METABOLISM_OF_LIPIDS_AND_LIPOPROTEINS	0.0445	0.0100
REACTOME_APC_C_CDC20_MEDIATED_DEGRADATION_OF_MITOTIC_PROTEINS	0.0129	0.0081
REACTOME_ACTIVATED_TLR4_SIGNALLING	0.0129	0.0055

In HD data set, seven pathways known to be related to HD documented in KEGG disease pathway are first examined (hsa05016). “KEGG_CALCIUM_SIGNALING_PATHWAY,” “KEGG_OXIDATIVE_PHOSPHORYLATION,” “KEGG_PROTEASOME,” “KEGG_APOPTOSIS” are identified by both methods where JointNormKS demonstrated on average better statistical significance. It worth noting that one HD-related pathway, “KEGG_RNA_POLYMERASE” is exclusively identified by JointNormKS. The full result of these HD related pathways is summarized in Additional File 4A. Furthermore, among the 57 gene sets exclusively identified by JointNormKS, we are surprised to find many cancer-related pathways. A further literature search shows that biological processes such as cell cycle, DNA repair, apoptosis and kinase signaling are both implicated in both diseases suggesting a potential link between two diseases (Plun-Favreau et al., 2010; Driver, 2012). The full list of enriched gene sets identified in HD are summarized in Additional File 4B.

## Conclusions and Discussion

In this study, we proposed two novel joint gene set enrichment analysis frameworks: JointNormKS and JointPathway aiming at borrowing shared information across similar disease from gene-level and pathway-level, respectively. Compared our previously developed joint gene analysis framework, the framework proposed here focused on pathway-level detection and demonstrated that assumption of similar disease sharing similar pathways is valid. The framework provides researchers with new opportunities to view their data from a different angle and could complement the limitation of gene-level analysis.

The two frameworks were first tested through simulation test and compared with MAPE, the current meta-analysis methods of gene set enrichment analysis. The results showed that the JointNormKS performed best among all tested methods under all simulation scenarios. The JointNormKS was then applied to two real data sets and identified a comparable or more number of enriched gene sets than analyzing the data set alone. Further examination revealed that JointNormKS could recover most of enriched gene sets that was identified by single data set analysis and the enriched gene sets exclusively identified by JointNormKS were mostly related to the disease. These results demonstrate that when similar diseases are jointly analyzed, the proposed joint gene set framework could borrow information from each other and improve identification power.

In the simulation test, we observed that in Scenario 1, the JointNormKS was not sensitive to the DE gene similarity (Figure 2). The reason is that after the joint analysis at gene-level, the rank of genes which are DE in both data sets would be prioritized to the top of the gene list ordered by posterior probability of DE status and the improvement of the rank of these genes is similar across different DE gene similarity values. Since the Normalized KS statistic is rank-sensitive, the ranks of enriched pathways would remain the same and so is the ROC although the posterior probability of these DE genes within an enriched pathway keep increasing. In scenario 2, when an enriched gene set in both data sets is composed of non-overlapped DE genes across two data sets, we observed that JointNormKS was still able to detect these gene sets and even had a better AUC improvement. The reason is that after gene-level joint analysis, the ranks of DE genes in the disease to be borrowed from would improve and Normalized KS statistic which is sensitive to these changes would increase the rank of these shared pathways. This might raise a concern whether this will lead to increased number of false positives. We would like to argue that the whole framework is designed based on the assumption that similar diseases would share similar enriched pathways. If this assumption holds, the JointNormKS framework would work well as demonstrated in simulation tests.

Three improvements need to be implemented in the future work. The first improvement is to design a likelihood test to detect the shared DE gene or enriched pathway similarity before joint analysis is performed so that researchers using this framework would have a better sense of whether these disease data sets should be jointly analyzed or not. The test procedure would be similar to that described in Chung et al. (2014). The second improvement is the ability of the framework to include more disease data sets to borrow as currently the size of prior probability vector increases exponentially based on the total number N of data sets (2^N^). A heuristic approximation or a hierarchical structure could be implemented as described in Lai et al. (2017). The third improvement is the incorporation of gene set dependence in the joint gene set enrichment analysis framework. In this study, gene set independence is assumed even many gene sets share common genes. This is hardly the case in real world. How to address the gene set/pathway dependence has been discussed and is a hot topic in the field of statistics (Tamayo et al., 2016; Tomoiaga et al., 2016; Xie et al., 2017). Extra work is needed to include it in the framework proposed in this study and several options would be explored in the future.

## Author Contributions

WQ and HL conceived and designed the study. WQ and HL developed the method, XW and HZ helped in method development. WQ wrote the computer program, analyzed data and interpreted the results. WQ, XW, HZ, and HL wrote the manuscript. All authors read and approved the final manuscript.

### Conflict of Interest Statement

The authors declare that the research was conducted in the absence of any commercial or financial relationships that could be construed as a potential conflict of interest.
